# Responses to COVID-19, small and medium enterprises’ corporate social responsibility and psychological capital of employees: From the mediating perspective of affective commitment

**DOI:** 10.1016/j.heliyon.2023.e15004

**Published:** 2023-03-30

**Authors:** Muhammad Farhan Jalil, Bilal Tariq, Muhammad Asif Zaheer, Zeeshan Ahmed

**Affiliations:** a School of Business and Management, University of Technology Sarawak, Malaysia; b Department of Management Sciences, COMSATS University Islamabad, Vehari Campus, Pakistan; c Institute of Management Sciences, Pir Mehar Ali Shah – Arid Agriculture University Rawalpindi, Pakistan; d Lahore Business School, University of Lahore Gujrat Campus, Gujrat, Punjab 50700, Pakistan

**Keywords:** Corporate social responsibility, Psychological capital, Affective commitment, Small and medium enterprises, COVID-19

## Abstract

The influence of corporate social responsibility (CSR) on employees' positive psychological capital in stressful situations remains unexplored in the literature for small and medium enterprises (SMEs). This study aims to examine how CSR could assist employees in developing psychological capital during the COVID-19 pandemic. In order to understand the aforesaid relationship, an attempt is made to study the mediation effect of affective commitment. Structural equation modelling (AMOS 21.0) was used for data analysis and hypothesis testing on a sample of 545 employees from 356 Malaysian SMEs. The results of this study showed that SMEs' CSR policies helped to improve the positive psychological capital of their employees during the COVID-19 pandemic. Also, affective commitment complementary mediates the relationship between CSR and psychological capital. During the COVID-19 pandemic, SMEs should come up with a consistent way to implement CSR policies and procedures to improve employees' psychological capital and increase their affective commitment toward the enterprise. There are two main contributions to the literature from this study, in addition to enriching previous empirical research on CSR. As a first contribution to the CSR literature, it examines how CSR impacts employees' psychological capital during a pandemic. COVID-19 is one of the recent pandemics that offers an opportunity to examine its effects on employee psychological state. Secondly, the results of the study add to the growing body of empirical research that supports affective commitment's significant relationship with CSR and enhances employees' psychological capital during a pandemic in a developing market.

## 1 Introduction

Worldwide, the Coronavirus-2019 (also known as COVID-19) pandemic has spread [1]. It has caused a severe economic crisis in which businesses, employees, and the economy have all been adversely affected [2,3]. Globally, approximately 665 million people were infected with COVID-19 b y December 2022, and more than 6.71 million of them died [4]. In response to the COVID-19 outbreak, many regions have become isolated, and economies have been negatively affected [5], which has adversely affected the global financial system as well [6]. In addition to disrupting global markets, it has changed the way employees, consumers, and organizations interact. It has also changed operational and managerial practices in small and medium enterprises (SMEs) [7].

COVID-19 introduces a completely new pandemic scenario giving us an insight into human behavior during a global pandemic where people are unsure how to respond and have no clear point of reference [8]. Furthermore, according to Nair [9], the pandemic created a climate of uncertainty and dread among workers in small and medium enterprises (SMEs) in developing economies. Kholaif and Ming [10] describe that SMEs are more likely to be hurt by the pandemic because they are made up of traditional industries that mostly hire people on a daily wage. Researchers (like [11,12]) have also identified that COVID-19 has a huge impact on business failures and employment, with SMEs having the most jobs at risk.

In this study, the corporate social responsibility (CSR) is considered as a management strategic tool and practice used by SMEs in crises. This can help to lessen crisis damage [13], gain employee affection [14], transform SMEs' images [15], and also help to achieve sustainability [16]. Therefore, SMEs' CSR policies would be closely scrutinized, particularly during an outbreak of COVID-19. CSR refers to firm strategies and determinations aimed at achieving long-term sustainable growth [17]. Following the outbreak of COVID-19, Malaysian SMEs did everything possible to assist in the battle against the disease [18]. According to Mao et al. [19], several businesses pooled their money to buy gauze masks, glasses, and other protective gear for front-line employees.

Previous research has shown that incorporating employees' perspectives into CSR can provide substantial benefits in numerous ways. These perspectives, among others, increase work satisfaction [20], safety behavior [21], organizational citizenship behavior [22], employee engagement [23], organizational identity [24], and creativity and innovation [8]. CSR has been found to enhance employees' psychological capital, particularly during times of crisis [19]. However, there is no clear understanding of the mechanisms involved [25]. From a SME point of view, besides their formal activities, employees are also required to undertake various informal tasks to enhance their psychological state at work [26]. This is to enable them to deal with an uncertain situation and achieve sustainable career growth [27]. Psychological capital is a valuable resource for individuals who are actively growing [28]. Moreover, Luthans et al. [29] categorized an optimistic psychological state as resilience, hope, optimism, and self-efficacy. Employee dedication and effectiveness have been demonstrated to be prompted by psychological capital [30]. Malaysian SMEs are required to place emphasis on developing the psychological capital of their employees [31], by fostering hope, resilience, self-efficacy, and optimism during the unprecedented fight against COVID-19.

Previous research [32,33] examined the psychological capital as a unified notion. According to Luthans et al. [34], positive psychological capital is linked to a variety of resources, including hope, resilience, self-efficacy, and optimism. Understanding the various influences of CSR on positive psychological capital makes it effortless to provide customized solutions for employee psychological rehabilitation [35]. Furthermore, it seems questionable whether CSR influences psychological capital [36]. During the COVID-19 epidemic, employees' positive psychological capital was severely reduced [19]. Further research on how CSR affects psychological capital will be beneficial not just to employees' ability to respond but also to the restoration of usual circumstances [8].

Our study investigated the psychological capital as a way to improve career interventions, focusing on the relationship between CSR and positive psychological capital, since managers/owners need to create conditions in which employees can foster vocational psychological development during difficult times [37]. Therefore, SMEs should consider CSR as an imperative component of enhancing employees' psychological capital [36]. To this end, “*the first objective of this study is* to determine the influence of CSR on psychological capital of SME employees during the COVID-19 pandemic in Malaysia.”

Previous studies [such as 19; 25; 36] highlighted a gap in the literature by examining the relationship between CSR and vocational behavior outcomes, which led researchers to clarify the reasons for this relationship. Particularly, the influential relationship between CSR and employees' psychological capital and the mechanisms through which it mediates have been little studied.

Recent studies [[38], [39], [40]] focus on the psychological capital in relation to employee attitude, behavior, and performance at workplaces. In recent research by Papacharalampous and Papadimitriou [25], employees' favorable behavior and attitudes in the workplace are said to be based on psychological capital. Moreover, the research of Mao et al. [19] explains that positive innovative behavior, active behavior, and positive employee attitudes like job satisfaction and organizational commitment are all components of positive psychological capital. Employees' negative behaviors and attitudes, such as job-quit intentions may be reduced as a consequence of positive psychological capital [41]. It is known that optimistic psychological capital can enhance employee commitment [33] because it is composed of mechanisms that drive employee behavior.

Moreover, the organizational commitment is critical in encouraging the individual recovery of positive psychological capital in a crisis [8]. Human resource practices such as psychological capital keeps employees ethically committed to the firm [42]. Hence, affective commitment is a method of managing emotions and achieving psychological rehabilitation in which individuals reflect on their prior experiences [43]. During the COVID-19 crisis, firms and employees must assess the role that affective commitment plays as a mediator in SMEs. This will help SMEs to regain their psychological capital.

According to Khaskheli et al. [22], when employees consider that their company practices CSR and that customers, society, and the environment benefit from such initiatives, their level of commitment improves. In addition, the existing literature on CSR offers little insight into the mechanisms that explain how CSR affects affective commitment at the individual level, particularly during crises such as the COVID-19 pandemic [8]. Scholars (such as [44,45]) have previously observed that affective commitment influences vocational behavior positively. Furthermore, Ribeiro et al. [33] noted that affective commitment is an important component of positive psychological capital and could be studied as a mediator between CSR and optimistic behavior in the future. Therefore, “*second objective of the study is* to determine the mediating effect of affective commitment between CSR and *psychological capital of SME employees during the COVID-19 pandemic in Malaysia”.*

Due to the lack of research in developing markets [19,25,33], this study focuses on Malaysia. To investigate the influential relationship between CSR and employees' psychological capital in the SME sector, as well as to investigate the role of affective commitment in mediating the relationship between CSR and workers' psychological capital in a developing market during the COVID-19 pandemic, the research is anticipated to answer the following two research questions (RQs): “*RQ1. How are CSR associated with employees’ psychological capital of SME employees during the COVID-19 pandemic? RQ2. To what extent does the affective commitment mediate the relationship between CSR and psychological capital of SME employees during the COVID-19 pandemic?*”

As a consequence of the COVID-19 pandemic, SMEs in emerging markets faced macro- and/or micro-level sustainability crises, but there has been limited empirical research on the significance of CSR in enterprises during pandemics. To that end, the study adapts social exchange theory (SET) to empirically assess the influential relationship between the constructs. We collected the data from SME employees in Malaysia and tested the conceptual model to address the following research gaps: First, there is scant research on how CSR influences the employees' psychological capital in the context of the COVID-19 pandemic. Therefore, the findings of the study explore the influential relationship between the variables within a SME context. Second, since the synergistic effects of CSR on employee psychological capital have not been empirically investigated, the research evaluated the mediating role of affective commitment. The findings address the unique relationship between CSR-affective commitment-psychological capitals among employees of SMEs in Malaysia during the COVID-19 pandemic. Third, in developing markets, there is a lack of empirical studies to identify the influential role of SME CSR during the COVID-19 pandemic. Thus, the findings of this study discovered the contemporary phenomena in Malaysia during the pandemic.

## 2 Literature review and hypotheses development

### 2.1 Concept of micro, small, and medium enterprises (MSMEs)

Generally, there seems to be no collectively putative definition of micro, small, and medium enterprises (MSMEs), and even most scholars have developed their respective definitions based on their study interests. Ireland et al. [46] defined enterprises that generate revenue through stimulating the economy by putting together unique resources and capabilities to capitalize on market possibilities. MSMEs have been defined by researchers and practitioners in the field of international business based on each country's socioeconomic development. In the United States, MSMEs are defined as businesses with 500 or fewer employees [47], whereas in Taiwan, MSMEs are defined as businesses with 650 or fewer employees [48]. MSEs, like in other countries had an important economic and social role in Malaysia, but there was no precise definition to discuss. Table 1 lists the SME Crop Malaysia descriptions of MSMEs [49].

**Table 1:** Classification adopted by SME Crop on MSMEs in Malaysia.

Enterprise level	Number of employees	Worth in million (RM)
Micro	below 5	Below 0.25
Small	From 5 to 50	From 0.25 to 10
Medium	From 51 to 150	From 10 to 25

### 2.2 Theoretical background

Social exchange theory (SET) was introduced by American sociologist George Homans, who developed a framework based on basic economics and behaviorism. Homans [50] stated that “an individual's social behavior is an exchange of goods involving both material and non-material ones such as symbols of approval or prestige” (p. 606). Furthermore, Rupp and Mallory [51] suggest that “SET is a key theory in CSR research.” In this study, SET was used to examine the influential relationship between CSR, employees' psychological capital, and affective commitment in the Malaysian SME sector during the COVID-19 pandemic. The recent study by Jia et al. [52] described that employees and firms are encouraged to engage in social exchange through CSR. According to Subba and Rao [53], the purpose of CSR is to promote well-being of employees and other stakeholders beyond economic and legal interests, which may encourage members to provide some benefits in return. In addition to meeting employees' needs, earning their trust, and elevating their dedication, firms' CSR practices help to build an optimistic association with them [54]. As previously stated (e.g. Refs. [52,55]), CSR practices help employees in considering appreciated and supported by their organization. The SET allows researchers to investigate how organizational activities affect employees' vocational behavior. SET has been used to study CSR practices. For instance, Subba and Rao [53] examine the impact of CSR on compassion at work, and Lee [56] investigates how CSR affects employee engagement. Therefore, it is appropriate to use SET in this study. This is because it also suggests that employees are obligated to provide benefits to stakeholders if they perceive the organization's actions as positive. Hence, the authors are able to conceptualize that the influence of CSR activities can enhance the psychological capital among employees, and motivate employees' affective commitment to organizations during the COVID-19 pandemic.

### 2.3 CSR and psychology capital

Kim et al. [57] describe CSR as a firm's ongoing commitment to socially and environmentally responsible for business practices, the well-being of society and employees, and economic growth. According to Hur et al. [58], firms that prioritize CSR are more likely to foster an open, transparent, and trusting workplace where employees feel comfortable attaining an optimistic psychological state and are also rewarded for their efforts to improve the firm's products and services. Another study by Hur et al. [59] describes the multiplicity of CSR-beneficial outcomes that may stem from an inclusive and encouraging work environment including the creation of high-quality goods and services. In addition, Brammer et al. [60] state that CSR activities encourage workers to find meaning in their work which is helpful for their psychological health. In a similar vein, Glavas and Piderit [61] argue that CSR-focused firms are those that foster flexible and open work environments, allowing employees to develop positive commitment to their jobs and the organization as a whole.

SMEs can support economic development and productive and life-sustaining services through CSR [62]. Furthermore, SME CSR may have a significant impact on a wide range of stakeholders [63]. For SMEs, to achieve sustainable development, CSR is an imperative factor. According to past studies [[64], [65], [66]], SME owners and managers are prepared to spend constrained resources to maximize community welfare activities, and their attention to satisfy the prospects and necessities of stakeholders (such as local communities, customers, and employees) has a major influence on employee psychological behavior.

Glavas [67] states that “interactions across the domains of organizational psychology, human capital, and CSR are critical since CSR may affect employee vocational behavior in numerous channels and there are insufficient studies on relevant intermediaries.” Some researchers [[68], [69], [70]] argue for additional empirical studies on the effects of CSR on employee development implying that CSR can play a role in the development of human capital and ethical behavior. Papacharalampous and Papadimitriou [25] explain that psychology capital serves as a link between CSR and human resource development approaches. This is due to the fact that psychological capital may not only be used to better understand behaviors and attitudes in the workplace, but it can also help with human resource development.

According to Luthans [71], “the study and application of positively oriented human resource strengths and psychological capacities that can be measured, developed, and effectively managed for performance improvement” (p. 59). Moreover, Luthans et al. [34] found that the psychological capital is a favorable psychological state that an individual generates over time. Further, they identified four main dimensions of positive psychology, “1. Self-efficacy; belief in one's own capacity to perform in challenging situations, 2. Optimism; a positive attitude towards achieving success in the present and the future, 3. Hope; demonstrating tenacity in achieving goals and, when necessary, redirecting pathways to those goals to maximize the possibility of success. 4. Resilience; the ability to recover and succeed in the face of adversity.” Youssef and Luthans [72] correlated psychological capital with workplace outcomes and found that resilience, hope, optimism, and self-efficacy are some of the positive psychological qualities that make up the relatively high construct of psychological capital.

Mao et al. [19] investigated the impact of CSR on employees' psychological capital. They surveyed 430 employees in China's tourism industry and found that CSR positively affected employees' psychological capital during the COVID-19 pandemic. In a cross-sectional study including 260 employees from 23 Greek companies, Papacharalampous and Papadimitriou [25] discovered a significant relationship between employees' perceptions of CSR and psychological capital. Rizvi and Nabi [73] assessed the relationship between CSR and the psychological capital of employees in India during the COVID-19 pandemic. Data was gathered from 154 employees using a cross-sectional approach, and the authors concluded that CSR activities were positively associated with the employees' psychological capital. The study by Hazzaa et al. [37] explores the influence of employees' perceptions of CSR on their psychological capital. A cross-sectional survey was conducted to collect data from 120 employees across the United States. The results of the study identified that CSR has a significant impact on employees' psychological capital. Furthermore, to examine the influence of CSR on the psychological capital of employees, Leal et al. [74] collected data from 279 employees and found a significant impact.

Individuals' psychological capital is critical in the COVID-19 pandemic [75], as it allows them to engage in the fight against the virus's effects and resume their everyday work and living routines [19]. Thus, SMEs' response to the COVID-19 pandemic and organizational commitment may provide employees with more consistent psychological capital. Employees' trust in the firm's eagerness and ability, as well as its creative output, can be revived by efforts to resolve crisis problems. This can be done as part of a variety of programs and strategies. It put employees in a better psychological position to deal with challenges and minimize the impact of the pandemic. Therefore, we believe that CSR can impact employees' psychological capital in SMEs during the COVID-19 pandemic, and we propose the following hypotheses.

Hypothesis 1 CSR has a positive effect on the employees' psychological capital during COVID-19 pandemic.

Hypothesis 1a Self-efficacy is a significant component of employees' psychological capital.

Hypothesis 1b Optimism is a significant component of employees' psychological capital.

Hypothesis 1c Hope is a significant component of employees' psychological capital.

Hypothesis 1d Resilience is a significant component of employees' psychological capital.

### 2.4 CSR and affective commitment

While the origins of CSR may be traced back a few decades, the concept gained traction in the post-industrial period as the expanding societal power of organizations [76] and their responsibility to stakeholders became more widely discussed and argued [77]. Therefore, businesses all over the world have begun to grasp the strategic importance of CSR in ensuring their continued existence and development. Simultaneously, CSR research aims to clarify the significant beneficial effects it has on businesses. According to Zhu et al. [78], CSR increased the stakeholder satisfaction and improved the economic conditions. According to Aguinis and Glavas [79], “the concept of CSR comprises two main areas when it comes to stakeholder groups: internal and external CSR. Internal CSR focuses on an organization's internal stakeholder groups, including workers and shareholders. External CSR concerns stakeholders outside the company, such as the government and customers.”

In the SME sector, both external and internal stakeholders benefit from CSR, particularly during the COVID-19 pandemic crisis [80]. In terms of the consequences for internal stakeholders, the significant empirical evidence has emerged to suggest that CSR enhances the employee vocational behaviors. Positive psychological state among employees are widely known to increase organizational productivity and profitability while also making it more sustainable [81,82].

Scholars (such as [[83], [84], [85], [86]]) have concentrated their studies on organizational behavior, attitude, and commitment since they feel it has a significant impact on the productivity of an industry. As Jalil et al. [85] state, “firm commitment” is “a psychological condition that describes the employee's relationship with the organization and has ramifications for the decision to continue or cease participation in the organization.” Moreover, previous research by Suliman and Iles [87] shows that “firm commitment is a three-dimensional concept: affective commitment, continuance commitment, and normative commitment.” Researchers such as Tett and Meyer [88] have placed a strong focus on affective commitment when studying employee relationships in the workplace.

Organizational commitment, according to Porter et al. [89], is “a strong bond between an employee and the company they work for, based on three factors: first, a strong belief in and acceptance of the company's goals and values; second, a willingness to put forth significant effort on behalf of the company; and third, a strong desire to remain a member of the company” (p. 604). Furthermore, Iverson and Buttigieg [90] state that elevated concentrations of affective commitment are linked to improve job performance, decreased absenteeism, reduced turnover, improved employee satisfaction, and higher work involvement.

Recent studies (such as [[91], [92], [93], [94], [95]]) have found that CSR has a favorable influence on affective commitment. Ahmad et al. [91] collected data from 736 academic employees in different universities in Pakistan to examine the relationships among CSR and affective commitment. The findings suggested that the employees' perceived CSR influences affective commitment. Ditlev-Simonsen [92] investigated the influence of CSR on employees' affective commitment. The author collected data from 512 employees of Scandinavian, Norwegian, and Swedish companies, and the results showed that employees' perceptions of CSR have a significant impact on their affective commitment. Bouraoui et al. [93] collected data from 161 private and public sector employees in Tunisia, and the findings of the study revealed the positive influence of CSR and employees' affective commitment. The purpose of Engizek and Yasin's [94] study is to investigate the relationship between CSR and employees' affective commitment. They collected the data from 522 employees in Turkey and found a positive impact of CSR on employees' affective commitment. Story and Castanheira [95] collected data from 190 employees and discovered that CSR and affective commitment had a positive relationship. Therefore, this research focused on the link between CSR and affective commitment in SMEs during the COVID-19 crisis. For instance, Low et al. [31] looked at the effect of CSR on affective commitment in Malaysian SMEs. In the SME sector, Harness et al. [96] investigated the impacts of employee perceptions of CSR on affective commitment. Therefore, the study recommends the following hypothesis:

Hypothesis 2 CSR has a significant impact on affective commitment during COVID-19 pandemic.

### 2.5 Affective commitment and psychological capital

According to van Waeyenberg et al. [97], “affective commitment” occurs when an individual's values and goals align with the organization. It refers to how emotionally committed and involved people are in their organizations [98]. The conservation of resources theory proposed by Hobfoll [99] can help us better understand the link between affective commitment and psychological capital. According to Hobfoll [99], individuals seek to capitalize on and preserve resources that are precious to them. An employee's emotional connection to the organization depends on their psychological competence, which implies resilience, hope, optimism, and self-efficacy [99].

According to growing evidence from research, positive psychological skills and abilities have been shown to have a positive influence on a variety of employment outcomes including affective commitment. Recent research (such as [33,43,100,101]) has found a correlation between affective commitment and individual psychological capital. Simons and Buitendach [100] investigated the links between work engagement, psychological capital, and firm commitment. Mangundjaya and Mufidah [43] examined the positive relationship between affective commitment and psychological capital. Similarly, Ribeiro et al. [33] focused on how psychological capital improves affective well-being, which has been associated with emotional commitment. According to Clarence et al. [101], the psychological capital has also been linked to affective commitment. Therefore, the research recommends the following hypothesis:

Hypothesis 3 Affective commitment has a significant impact on the employees' psychological capital during COVID-19 pandemic.

### 2.6 CSR, affective commitment, and psychological capital

Although CSR increases employee commitment, there is currently less evidence of a fundamental association between CSR engagement, behavior, and attitudes at the workplace. The findings of Ahmad et al. [91], who surveyed 736 employees from different institutes of higher education, and found that the implications of CSR initiatives can enhance the levels of employee commitment.

Furthermore, CSR has the potential to improve employee self-identity and build positive psychological distinctions. Oo et al. [102] found that CSR involvement strengthens the awareness and self-esteem of workers when viewed through the lens of psychological process and individualistic method. Since there is a natural overlap between learning strategies for personal growth and psychological capital, these ideas are put under the CSR flag. Therefore, they promote individuality even as they progress and flourish inside the affirmative working variables and result in favorable behaviors and attitudes [19]. Workers' favorable perceptions of organizational CSR are therefore likely to contribute to a rise in psychological capital and the creation of a self-perpetuating vicious circle that might improve employees' affective commitment [25].

Based on CSR literature, the research of Khaskheli et al. [22] was chosen to assess the mediating role of affective commitment within the context of CSR. The results show that affective commitment mediates the association between workers' perceptions of CSR and perceived firm attitudes and behaviors. After evaluating CSR-related research, Papacharalampous and Papadimitriou [25] stated that the psychological capital can be a powerful indication of organizational commitment. Despite the study's findings, there is still a lot of uncertainty about the importance of psychological capital. Previous studies have not been able to explain in a clear and practical way how CSR affects employees' psychological capital through affective commitment. Based on the previous discussion, this research includes affective commitment as a “mediating construct” in the relationship between CSR and psychological capital during the COVID-19 pandemic. Thus, the following hypothesis is proposed:

Hypothesis 4 Affective commitment mediates the relationship between the CSR and psychological capital during COVID-19 pandemic.

### 2.7 Conceptual framework

**Fig. 1:**
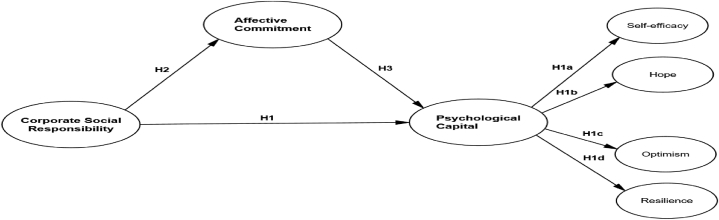
Conceptual framework. Source: Adapted from Mao et al. (2021).

In this study, SET is used to develop a conceptual framework (see Fig. 1). The proposed conceptual framework is able to improve employees' psychological capital, their perception of the value of CSR, and their affective commitment to resolving the issue of the COVID-19 pandemic.

## 3 Methods

### 3.1 Data collection: procedure and sample

A quantitative approach was used in this research to evaluate the hypotheses and to meet the research objectives. Due to the COVID-19 outbreak and travel limitations, a self-administered questionnaire and an online questionnaire approach were used to collect data.

The respondents in this study were employees of SMEs, and data was collected from five cities of Malaysia: Kuala Lumpur, Kuching, Kota Kinabalu, Malacca, and Kota Bharu. In particular, we used stratified random sampling to select participants from the 356 SMEs listed by SME Crop Malaysia. Chin and Lim [103] stated that “the services and manufacturing sectors have always dominated, accounting for 90.6% of Malaysian SMEs and contributing 83.3% to overall GDP.” Therefore, this study focused on two strata of SMEs: the manufacturing sector and the service sector. According to the SME Crop Malaysia Annual Report 2022 49, the total number of service sector employees is approximately 5000, while the manufacturing sector is around 3000. To obtain samples from each stratum, Krejcie and Morgan's [104] criteria were used (sample size for service sector = 357 and manufacturing sector = 341). Hence, we distributed a total of 698 questionnaires to the respondents, but only 545 surveys were completed, yielding a response rate of 78.1%. According to Tanaka [105] and Kline [106], “a study using structural equation modelling (SEM) uses the smallest sample size of 200 cases.” At this point, the sample size of this study is suitable for further investigation.

### 3.2 Measurement of variables

For this research, the data was gathered utilizing adapted questionnaires from earlier studies in order to examine the influence of CSR on employees' psychological capital through the mediating effect of affective commitment in the relationship. The questions were rated on a 5-point Likert scale, with 1 being “strongly disagree” and 5 being “strongly agree.” The questions were adapted to measure the influence of CSR from Zhang et al. [21]. The items to measure employees’ psychological capital (resilience, hope, optimism, and self-efficacy) were modified from Jalil et al. [107]. To measure the mediating effect of affective commitment, questions were modified from Mohammed et al. [8].

### 3.3 Statistical analysis

The data was analyzed using structural equation modelling (SEM) to evaluate the hypothesis. A maximum likelihood approach in SEM is used to evaluate the structural and measurement models [108]. The convergent validity and causal link between modified items, and variables in the measurement model were tested using confirmatory factor analysis (CFA) [109]. A structural model, on the other hand, is used to examine the link between exogenous and endogenous factors [110]. The AMOS 21.0 software SEM analysis tool was used in this study, and according to Kline [111], it requires a minimum sample size of 100–200 respondents to build an acceptable model.

## 4 Results

### 4.1 Demographic characteristics

In this study, 545 employees from 356 SMEs were surveyed. Table 2 presents the statistical findings of demographic characteristics based on questionnaire items (such as gender, age, marital status, education, ethnic group, religion, enterprise level, enterprise activities, position in the enterprise, and income level) to assess the participants' backgrounds.

**Table 2:** Demographic profile of respondents.

Constructs		Number	Percentage
Gender	Male	394	72.3%
	Female	151	27.7%
Age	Below 20	6	1.1%
	20–30	182	33.4%
	31–40	166	30.5%
	41–50	131	24.0%
	51 and above	60	11.0%
Marital status	Single	134	24.6%
	Married	363	66.6%
	Widow	25	4.6%
	Divorced	23	4.2%
Education	Diploma or high school or less	215	39.4%
	Bachelors	189	34.7%
	Masters	105	19.3%
	Doctorate	33	6.1%
	Other	3	0.5%
Ethnic group	Malay	277	50.8%
	Chinese	168	30.9%
	Indians	77	14.1%
	Others	23	4.2%
Religion	Muslim	304	55.8%
	Hindu	54	9.9%
	Christian	78	14.3%
	Buddhist	88	16.1%
	Others	21	3.9%
Enterprises level (based on number of employees, N = 356)	Small enterprise	218	61.2%
	Medium enterprise	138	38.8%
Enterprises activities (N = 356)	Manufacturing sector	123	34.6%
	Services sector	233	65.4%
Position in the enterprise	Lower level executives	269	49.4%
	Middle level executives	175	32.1%
	Upper level executives	101	18.5%
Income level	Less than RM3000	124	22.8%
	RM3000-4000	167	30.6%
	RM4001-5000	138	25.3%
	RM 5001 and above	116	21.3%

Note: N = number of selected SMEs.

### 4.2 Normality statistics

The most crucial assumption in SEM is multivariate normality testing [112]. According to Hopkins and Weeks [113], a variable with skewness and kurtosis values in the ±3 range is considered appropriately distributed. The statistical values of skewness and kurtosis for each construct were calculated in this research and are listed in Table 3.

**Table 3:** Descriptive statistics.

Variables	Likert scale	Mean	Std. Dev.	Skewness	Kurtosis
CSR	1–5	3.75	0.52	0.821	0.044
Affective commitment	1–5	3.46	0.61	−0.663	−0.463
Self-efficacy	1–5	4.12	0.56	0.105	−0.5.25
Hope	1–5	4.04	0.45	0.145	−0.076
Optimism	1–5	3.92	0.41	0.771	0.098
Resilience	1–5	3.65	0.48	−0.173	0.822

### 4.3 Reliability and discriminant validity

According to Taber [114], a coefficient alpha of 0.7 or higher is acceptable, while 0.8 or above is regarded as having outstanding internal consistency. In this study, CSR had a coefficient alpha (α) of 0.878, self-efficacy had α of 0.894, hope had α of 0.923, optimism had α of 0.866, and resilience had α of 0.842. The coefficient alpha for affective commitment as a mediating component is 0.904.

Correlation is used to determine the link between the variables [115]. A Pearson correlation analysis is used in this study to assess the correlation between all of the factors. Table 4 shows the correlation analysis results for all of the variables considered in this study.

**Table 4:** The correlation of the constructs.

	1	2	3	4	5	6
CSR	1					
Affective commitment	0.345	1				
Self-efficacy	0.451	0.327	1			
Hope	0.432	0.468	0.312	1		
Optimism	0.482	0.377	0.368	0.415	1	
Resilience	0.370	0.398	0.316	0.458	0.384	1

### 4.4 Assessment of confirmatory factor analysis (CFA)

**Fig. 2:**
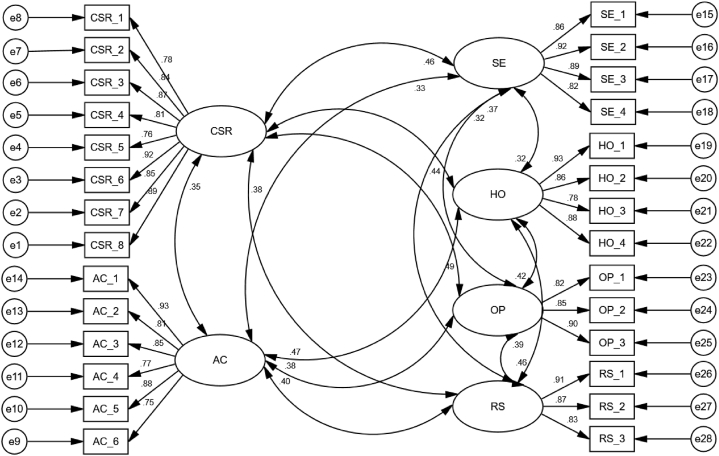
Overall measurement model. Note: CSR (corporate social responsibility), SE (self-efficacy), HO (hope), OP (optimism), RE (resilience), AC (affective commitment).

Confirmatory factor analysis (CFA) is the first stage of the SEM statistical method and it allows the researcher to evaluate the fitness between observed data [109]. Fig. 2 shows a measurement model with satisfactory factor loading (above 0.70) and acceptable fit indices (such as RMSEA = 0.054; chi square = 631.462; df = 544; GFI = 0.916; AGFI = 0.927; CFI = 0.932; CMIN/df = 1.361) as identified by Widaman and Thompson [116].

In SEM, CFA is frequently used to assess construct validity [109]. Convergence validity was used in this study to examine the data validity using average variance extraction (AVE) and composite reliability (CR). CR is higher than 0.6 in general, implying that all measurement items are more consistent [117]. By establishing whether the AVE is greater than 0.5, the measurement items may better describe the features of each research variable in the model [118]. The CR and AVE of all constructs employed in this investigation are over the threshold, as indicated in Table 5.

**Table 5:** Convergent validity evaluation.

Items	Measurement Path	FL	CR	AVE
CSR				
CSR_1	During the COVID-19 pandemic, the firm is trying to fulfil its philanthropic social responsibility.	.78	.946	.708
CSR_2	During the COVID-19 pandemic, the firm is trying to meet government strategies.	.84		
CSR_3	During the COVID-19 pandemic, the firm is trying to meet requests for NGOs to bettering local communities.	.87		
CSR_4	During the COVID-19 pandemic, the firm is actively providing money and materials.	.81		
CSR_5	During the COVID-19 pandemic, the firm is trying to assist both internal and external stakeholders.	.76		
CSR_6	During the COVID-19 pandemic, the firm is attempting to enhance the quality of its services.	.92		
CSR_7	During the COVID-19 pandemic, the firm is helping to improve employee health.	.85		
CSR_8	During the COVID-19 pandemic, the firm is attempting to establish a socially responsible reputation.	.89		
**Affective commitment**				
AC_1	I would be delighted to work for this firm for the remainder of my career.	.93	.932	.696
AC_2	I feel as though this firm's troubles are my own, during the COVID-19 pandemic.	.81		
AC_3	I had the great sensation of “belonging” to this firm, during the COVID-19 pandemic.	.85		
AC_4	I became emotionally engaged with this firm, during the COVID-19 pandemic.	.77		
AC_5	I feel like I am “part of the family” at this firm, during the COVID-19 pandemic,	.88		
AC_6	During the COVID-19 pandemic, this firm had a lot of personal significance for me.	.75		
**Self-efficacy**				
SE_1	I am confident in my capability to contribute to the firm's strategy, during the COVID-19 pandemic.	.86	.928	.763
SE_2	I am confident in my ability to represent my job area to top manager/owner, during the COVID-19 pandemic.	.92		
SE_3	I am confident in my capability to support in the setting of objectives at my workplace, During the COVID-19 pandemic.	.89		
SE_4	I am comfortable approaching suppliers or customers to address issues, During the COVID-19 pandemic.	.82		
**Hope**				
HO_1	I am working hard to achieve my professional objectives, during the COVID-19 pandemic.	.93	.922	.747
HO_2	I could come up with a number of methods to get out of it, during the COVID-19 pandemic.	.86		
HO_3	I consider myself to be a pretty effective employee, during the COVID-19 pandemic.	.78		
HO_4	I can think of a number of approaches to achieving my present job objectives, during the COVID-19 pandemic.	.88		
**Optimism**				
OP_1	When things are unclear at work during the COVID-19 pandemic, I always hope for the best.	.82	.893	.735
OP_2	Despite the COVID-19 pandemic, I am optimistic about what will happen to me professionally in the future.	.85		
OP_3	During the COVID-19 pandemic, Things never turn out the way I'd like.	.90		
**Resilience**				
RS_1	At work, I frequently deal with problems in one form or another, during the COVID-19 pandemic.	.91	.904	.758
RS_2	At workplace, I believe I am capable of juggling several tasks, during the COVID-19 pandemic.	.87		
RS_3	I've been through difficult periods at work, I know how to get through them, during the COVID-19 pandemic.	.83		

Note: FL = factor loading, CR = composite reliability, AVE = average variance extraction.

### 4.5 Assessment of structural model

**Fig. 3:**
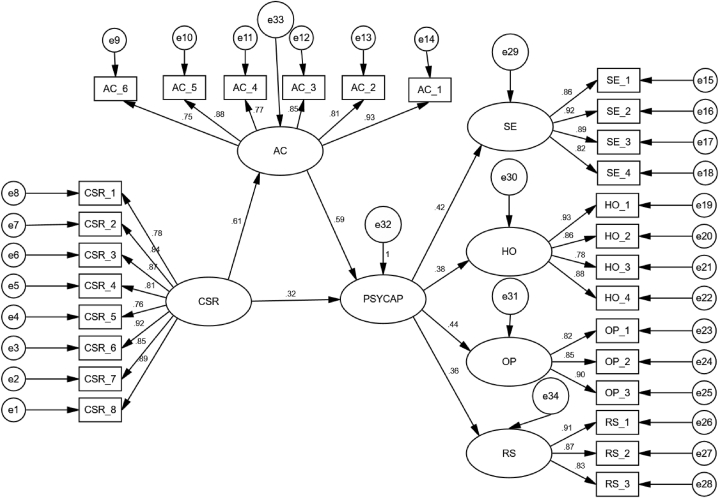
Structural model.

In the second stage, Boyle et al. [119] describe how a structural analysis was carried out to assess the relationship among the constructs. The structural model, as shown in Fig. 3, demonstrates conformity (Chi-square = 1.453; GFI = 0.944; AGFI = 0.932; CFI = 0.955; TLI = 0.951; RMSEA = 0.034), followed by the study of Kline [111], showing that the study's hypothesized model was supported.

As shown in Table 6, based on the findings, the hypotheses “H1, H1a, H1b, H1c, H1d, H2, and H3” were significant in evaluating the hypothesized association. Kline [111] noted that each of the study's hypotheses had substantial and well-supported standardized estimates.

**Table 6:** Testing direct relationship.

Hypothese and Paths	β	T-Value	Supported
H1: CSR → Psychological capital	.32**	4.372	Yes
H1a: Psychological capital → Self-efficacy	.42***	5.427	Yes
H1b: Psychological capital → Hope	.38***	5.018	Yes
H1c: Psychological capital → Optimism	.44***	5.589	Yes
H1d: Psychological capital → Resilience	.36***	4.732	Yes
H2: CSR → Affective commitment	.61***	6.195	Yes
H3: Affective commitment → Psychological capital	.59***	6.033	Yes

*p < 0.05, **p < 0.01, ***p < 0.001.

### 4.6 The mediation analysis

The study found that the affective commitment mediates the relationship between CSR and employees’ psychological capital. According to the complementary mediation recommendations by Fornell and Larcker [120] and Zhao et al. [121], Hypothesis 4 (H4) is accepted because all standardized path coefficients are significant and positive.

Based on the Jalil et al. (2021a) 107 study, we also used the bootstrapping approach to confirm the study's findings and found that complementary mediation had occurred. This study was done using the Maximum Likelihood Bootstrapping method with a bias correction confidence interval of 95% and a bootstrap sample size of 1000, as shown in Table 7.

**Table 7:** Bootstrapping results.

Hypothesis	Results	Mediation
H4: CSR → Affective commitment →Psychological capital	Path 1: β = .62*** t-value = 6.345; Path 2: β = .58*** t-value = 5.932	Complementary mediation

## 5 Discussion, implications, and recommendations

### 5.1 Discussion

The influence of SME's CSR on the psychological capital of employees was investigated against the backdrop of the COVID-19 pandemic. Positive psychological capital, including resilience, hope, optimism, and self-efficacy, was effectively strengthened by CSR using SME COVID-19 responses. By examining the methods adopted by SMEs to improve employee's psychological conditions, the focus of this research is to give theoretical and practical insights on how to better deal with the psychological disturbance caused by the epidemic.

Employee psychological capital benefits from SME CSR. Employee engagement in enterprise COVID-19 responses increased as the SME initiated more CSR. When faced with difficult tasks, employees have the confidence to perform better and the ability to create the required activities to attain achievement. Hence, the first aim of the research was “to determine the influence of CSR on psychological capital of SME employees during the COVID-19 pandemic in Malaysia”.

The first aim was to test Hypothesis 1 which states that CSR has a significant influence on the psychological capital. As a result, the positive and significant association between them is confirmed by the path from “CSR to psychological capital of 0.32, t-value 4.372, and p-value <0.05”. The study's findings are consistent with previous research by Mao et al. [19] and Hazzaa et al. [37]. According to the findings of this study, the more assistance a company provides to combat COVID-19, the greater the psychological capital of its employees, and the more options available to workforces to deal with COVID-19 and related issues. Effective organizational strategies, according to Aguinis et al. [122], may help employees to overcome hardship and pursue improvement. Hence, this study suggests that when a pandemic strikes CSR is critical in ensuring that employees stay on the job and heal from the psychological trauma caused by the outbreak. Moreover, according to Zhou et al. [123] and He et al. [124], firm approaches can improve psychological capital. Similarly, this study found that during a pandemic, SME CSR sends out positive vibes to employees. This encourages them to feel that the pandemic and challenges are temporary and that they can be overcome through collaborative efforts. This increases the psychological capital of employees.

Moreover, the first research objective revealed that resilience, hope, optimism, and self-efficacy are determinants of psychological capital. Self-efficacy as a significant factor of psychological capital was shown to be predicted by Hypothesis H1a “(path 0.42, t-value 5.427, and p-value <0.05).” Hypothesis H1b, hope is a significant component of psychological capital, with a “path = 0.38, t-value = 5.018, and p-value <0.05.” This study tested hypothesis H1c, which suggests that optimism is a significant part of psychological capital. “The coefficient was 0.44, the t-value was 5.589, and the p-value was 0.05.” The hypothesis (hypothesis H1d) that resilience is a significant factor in the psychological capital path was tested. Resilience predicts positive psychological capital, as evidenced by a “path coefficient = 0.36, t-value = 4.732, and p-value <0.05.” Hence, the research reveals that the four predictors (resilience, hope, optimism, and self-efficacy) were all positive psychological capital, this is in line with the findings of Luthans et al. [125].

The intention of this research was to assess a model that identified affective commitment as a mediating construct in the association among employees' perceptions of their SME's CSR and psychological capital. Therefore, the second objective of the research was “*to determine the mediating effect of affective commitment between CSR and psychological capital of SME employees during the COVID-19 pandemic in Malaysia*”.

Based on the second objective of the study, Hypothesis 2: CSR has a positive effect on affective commitment. Hence, “the path from CSR to affective commitment was 0.61, t-value 6.195, and p-value <0.05” confirms the significant association between them. Hypothesis 3: Affective commitment has a positive effect on employees' psychological capital. Thus, “the path from affective commitment to psychological capital is 0.59, t-value 6.033, and p-value <0.05” confirms the significant association among affective commitment and psychological capital. Hypothesis 4: Affective commitment mediates the association among CSR and employees' psychological capital. The statistical results show that “all standardized path coefficients are significant and positive (CSR → affective commitment → PsyCap)”, confirming affective commitment's complementary mediating role among SME's CSR and psychological capital of employees.

According to the findings, employees' perceptions of CSR have a significant direct impact on their affective commitment and psychological capital. Furthermore, the psychological capital has a direct and considerable impact on affective commitment. Surprisingly, CSR views and psychological capital account for a significant percentage of the variation in employees' affective commitment, particularly in the COVID-19 epidemic. These results support prior research (such as [72,101]) indicating that employees with positive evaluations of their SME's CSR have a much greater level of affective commitment to the firm as well as a higher level of psychological capital. As a result, this intriguing finding suggests that the CSR actions taken by SMEs can have significant impacts, even in adverse circumstances. This may trigger the development of psychological capital in the workplace.

Our findings revealed the existence of a complementary mediation effect of affective commitment in the association between CSR perception and employees' psychological capital during the COVID-19 pandemic. This indicates that the higher-order concept of affective commitment is involved in the specific interaction, adding to our knowledge of how psychological capital is developed at work during a pandemic. Evidence of complementary mediation helps to understand the process through which an impact functions from a practical viewpoint. Our findings in this scenario indicate that affective commitment plays a significant role in the process by which CSR influences individual psychological capital across all employee groups. In a previous study, Leal et al. [74] showed the mediation function of affective commitment in the association between CSR and organizational citizenship behaviors. Our research fills up this knowledge gap by presenting additional empirical evidence of affective commitment's mediation role in the CSR perception-psychological capital relationship.

### 5.2 Theoretical implications

It is proposed that this study adds to the current literature on SET. In the context of an unusual pandemic, the employees' psychological capital was examined as a result of SME CSR. Employee's psychological capital in SMEs is typically important in enhancing job performance and healthy workplace behavior [19,126,127]. The impact of positive psychological capital has received a lot of attention in previous studies, but the retention and growth of psychological capital among employees have received less attention. Employees faced serious health risks due to the COVID-19 epidemic, as well as uncertainty regarding their earnings and job security. SME's, on the other hand, can help with the retention and recovery of psychological capital through suitable CSR policies and practices, under the unique conditions of COVID-19. As a result, the study's findings identified an influential relationship between CSR and employees' psychological capital in a Malaysian SME context during the COVID-19 pandemic. Moreover, the study found that maintaining employee's psychological capital in SMEs has become a key concern. In addition to their individual efforts, SME can help to preserve employee optimistic psychological capital (resilience, hope, optimism, and self-efficacy) through CSR practices.

Furthermore, the CSR-affective commitment-psychological capital model builds on previous research by looking at possible mediators in the association between SME's CSR and behavior and attitudes at work [[128], [129], [130]]. The findings of the study provide strong evidence that affective commitment serves as a complementary mediator in the process of developing psychological capital by SME CSR. This particular study gives insight into the ability of CSR to integrate affective commitment and workers' psychology, demonstrating the developing nature of psychological capital in the CSR context. Furthermore, it elucidates how the affective commitment part of CSR interacts with employees' positive psychological capital at work, suggesting that SMEs' CSR efforts may contribute to the development of human capital.

### 5.3 Practical implications

SMEs must actively accept social responsibility in a situation like COVID-19. CSR not only supports SMEs in establishing a favorable public image, but it also assists employees in maintaining a positive psychological condition. The COVID-19 pandemic devastated people's trust all across the world, especially among SME employees in Malaysia, where the repercussions were considerably more severe. It made employees more aware of the importance of paying attention to every move made by SMEs than they had been previously. In the face of a disaster like COVID-19, SMEs may considerably improve their public image by demonstrating social responsibility by caring for staff, showing genuine concern for consumers and distributors, and helping the local community cope. This may lead to improved staff satisfaction with COVID-19 reactions in the workplace. Furthermore, these efforts serve as a powerful leadership model for workers and others. As a result of these effortless activities, employees were more proud to be part of a helpful, compassionate, and generous SME. The CSR displayed by SMEs will serve as a solid platform for employee follow-up initiatives. In these conditions, SMEs should not be reluctant to properly communicate their social responsibility policies and practices.

Positive psychological capital is something that owners and managers must pay attention to resilience, hope, optimism, and self-efficacy. Companies that pay more attention to their employees' psychological capital may be able to help them return to normalcy faster. Employees of SMEs faced the extreme psychological stress during COVID-19 because of a slew of difficulties, including health and safety hazards, unstable earnings, cramped living standards and environments, and shifts in working time and practices. Employees must maintain a peaceful and balanced psychological condition in order to work and produce value for their employers. They must also grow their psychological capital reserves. CSR activities help employees believe more in their skills and abilities, which makes them more resilient. Furthermore, enterprises would take the lead to identify keys to difficulties in such instances, pursue further stable potential growth routes for the future, and acquire experience and knowledge for their long-term development. SME owners and managers should give direction to their employees on a regular basis, using data and case studies from the past. This will help them think and act in more meaningful ways about their current situation and their future. They should also recognize that current issues are transient. Employees' confidence must be strengthened, and it must be communicated that the SME sector has a promising future in the medium-to-long term.

By guiding and enhancing employees' ability to self-adjust, SMEs can help them maintain and regain healthy psychological conditions. Employee commitment has a favorable impact on the recovery of psychological capital. Because of the spread of COVID-19 and the challenges in assessing the authenticity of news and other people's perspectives, it was easy for people to experience a variety of negative emotions. SME must promote encouraging news and actions in order to foster a culture of working together to overcome challenges. They could use training or other learning events to divert employees' focus away from the pandemic. SME should assist employees in developing a reasonable and scientific assessment of the pandemic so that they may progressively get a better understanding of the situation through continual discussion. Through increased firm attention to psychological capital, the pandemic's negative psychological number of casualties can be decreased, and therefore employees' skills to cope with it can be improved.

In light of the above discussion, the findings of this study may play a crucial role in assisting SME managers/owners in accessing global crises and acquiring knowledge from the COVID-19 pandemic, as well as in guiding their activities or rethinking business strategies long after the pandemic has passed. These managers/owners must develop concrete CSR activities and develop targeted programs to help their organizations weather these storms and strategically heighten the awareness of their SMEs and employees. In times of economic uncertainty, our research shows that corporate social responsibility (CSR) policies, especially those that focus on ethics and giving back to the community, may have an impact on employees' affective commitment and psychological capital. In Malaysia, the Small and Medium Industries Development Corporation (SMIDEC) is responsible for formulating and enforcing all SME-related policies. With its emphasis on the importance of CSR policies and practices for SMEs, the study suggests that SMIDEC should prioritize these factors over the growth and survival of the sector.

### 5.4 Limitations and recommendations

First, the study's cross-sectional approach has limitations in analyzing the causal links between CSR, affective commitment, and psychological capital during the COVID-19 pandemic. Therefore, in the future, longitudinal research should be employed to uncover specific causal linkages. Second, due to the COVID-19 restrictions on travel, this study was limited to metropolitan cities in Malaysia. Future research should assess the effects of CSR on rural and urban employees' psychological capital in comparison. Third, relying on a single data source might easily result in source issues. CFA testing and anonymous questionnaire participation confirmed that this erroneous factor was within the acceptable limit. To limit the common mistake as much as possible, future studies should collect data from a variety of sources. Fourth, the focus of this study was on the impact of CSR on individual employees, rather than a firm-wide assessment. Future, studies should investigate a multi-level assessment and the variety of data sources. Finally, the empirical findings of this study revealed a complementary mediating effect of affective commitment between CSR and SME employees' psychological capital. While researchers can incorporate mediators or moderators into the established framework to obtain new results.

## Ethical approval

Approval was obtained from the University of Technology Sarawak Ethics Committee (UTS-EC). The procedures used in this study adhere to the tenets of the Declaration of Helsinki.

## Author contribution statement

Muhammad Farhan Jalil: Analyzed and interpreted the data; contributed reagents, materials, analysis tools or data; wrote the paper. Bilal Tariq: Analyzed and interpreted the data; wrote the paper. Muhammad Asif Zaheer: Contributed reagents, materials, analysis tools or data. Zeeshan Ahmed: Analyzed and interpreted the data.

## Funding statement

The authors received no financial support for the research, authorship, and/or publication of this article.

## Data availability statement

Data will be made available on request.

## Declaration of competing interest

The authors declare that they have no known competing financial interests or personal relationships that could have appeared to influence the work reported in this paper.
